# The Effects of Personalized Observation, Execution, and Mental Imagery (POEM) Therapy in Logopenic Primary Progressive Aphasia: A Telepractice-Based Single-Case Study

**DOI:** 10.3390/brainsci16060653

**Published:** 2026-06-20

**Authors:** Sandrine Basaglia-Pappas, Hina Solignac, Édith Durand

**Affiliations:** 1 Cognitive Psychology and Neuropsychology Department, University of Mons, 7000 Mons, Belgium; 2 Department of Speech-Language Pathology, University of Montpellier, 34000 Montpellier, France; hinasolignac.orthophoniste@gmail.com; 3 Department of Speech-Language Pathology, Université du Québec à Trois-Rivières, Trois-Rivières, QC G9A 5H7, Canada; edith.durand@uqtr.ca; 4 Centre for Interdisciplinarity Research in Rehabilitation of Greater Montreal (CRIR), Montreal, QC H3S 1M9, Canada

**Keywords:** primary progressive aphasia, verb anomia, speech and language therapy, telepractice

## Abstract

**Highlights:**

**What are the main findings?**

- Improvements in the frequency and quality of co-verbal gesture use were observed following POEM (Personalized Observation, Execution and Mental Imagery) therapy on a person with logopenic variant primary progressive aphasia, especially for untrained verbs, suggesting generalization of the gesture execution strategy.
- While the level of competence in verb naming remained stable, suggesting a prophylactic effect, performance in narrative discourse and functional communication measures improved, suggesting that POEM could have a positive effect on verb production in connected speech and functional communication.

**What are the implications of the main findings?**

- By encouraging the production of semantically congruent gestures, sensorimotor-based interventions such as POEM therapy may promote compensatory pathways for lexical access in logopenic variant primary progressive aphasia.
- The delayed treatment effect and gesture improvements suggest that the benefits of gesture-based interventions may emerge gradually over time. This finding highlights the potential importance of extended treatment duration and sustained practice in primary progressive aphasia.

**Abstract:**

**Background/Objectives:** Verb anomia is a common symptom of primary progressive aphasia (PPA), particularly in the logopenic variant (lvPPA). Despite the central role of verbs in sentence construction, interventions specifically targeting verb anomia remain scarce. This study examined the effects of Personalized Observation, Execution and Mental Imagery (POEM) therapy, grounded in evidence that sensorimotor systems are recruited during action verb processing and may support verb retrieval. **Methods:** A 74-year-old woman with lvPPA completed pre- and post-POEM assessments (linguistic, cognitive, thymic, and motor). POEM consisted of 15 telepractice sessions delivered three times weekly using a systematic procedure with three sensorimotor strategies: action observation, gesture execution, and mental imagery. **Results**: No significant gains were observed in verb production across naming tasks, spontaneous speech, or functional communication contexts. However, co-verbal gesture use increased in frequency and quality, particularly during the naming of untrained verbs, suggesting that the gesture execution strategy was generalized. A delayed treatment effect was also noted, raising questions regarding the optimal duration of POEM in neurodegenerative conditions. **Conclusions:** While no statistically significant improvements in verb production were observed, qualitative analyses revealed increased use and quality of co-verbal gestures, suggesting that POEM had a compensatory effect. Future research involving larger cohorts and longer periods could help clarify the benefits of POEM therapy. It would also be relevant to compare in-person and remote delivery formats to evaluate POEM therapy’s robustness and adaptability in different clinical contexts. To conclude, these findings remain preliminary and should be interpreted with caution, particularly given the lack of significant improvement in primary verb production outcomes.

## 1. Introduction

Primary progressive aphasia (PPA) refers to a group of neurodegenerative syndromes characterized by progressive impairment of speech and language abilities [1,2]. Current international consensus criteria distinguish three principal variants: the non-fluent/agrammatic variant (nfvPPA), the semantic variant (svPPA), and the logopenic variant (lvPPA) [3]. In the absence of disease-modifying treatments, the development of targeted, symptom-focused interventions, particularly speech and language therapy, remains crucial. Indeed, speech and language therapy constitutes a key component in the clinical management of PPA, aiming to slow down language decline and support the preservation of communicative abilities or compensation for their loss [4,5,6]. nfvPPA and svPPA are predominantly associated with frontotemporal lobar degeneration pathology, whereas lvPPA is most frequently linked to underlying Alzheimer’s disease neuropathology, marked by amyloid-β accumulation and tau-related neurofibrillary changes predominantly in temporoparietal regions, disrupting distributed language and phonological networks [7,8,9,10].

First identified in 2004 by Gorno-Tempini et al. [7,11], lvPPA is characterized by prominent anomia and impaired sentence repetition, reflecting deficits in phonological processing, often attributed to verbal working memory deficits [7]. Speech output is generally fluent but marked by frequent pauses and word-finding hesitations, but without frank agrammatism or early severe semantic impairment. People with lvPPA may also exhibit behavioral and affective changes, including anxiety and depressive symptoms [12,13]. Although nonlinguistic deficits are generally considered to emerge as the disease progresses, they have also been documented in the early stages. In this context, executive dysfunction has recently been reported to be an associated deficit [14,15].

Verb production, which involves a three-stage psycholinguistic process—conceptualization, formulation/lexicalization, and articulation [16,17]—constitutes a core organizing element of any utterance, as it encodes events and structures syntactic relations. Verbs are generally more vulnerable than nouns, especially in lvPPA, due to their more complex argument structure and their reliance on fronto-perisylvian networks. From a neuroanatomical perspective, verb production has been shown to involve the left superior temporal gyrus, the left prefrontal cortex, and the right superior parietal lobule [18,19,20]. The latter two regions in particular appear to be more strongly engaged during verb production than noun production [19]. Lesions affecting these areas, or the networks that connect them, may disrupt the sensorimotor representation of actions. Action imageability plays a critical role in verb representation, given the often abstract nature of action-denoting lexical items.

Given the central role of verbs in sentence structure [21,22] and the limited number of interventions specifically targeting verb anomia in lvPPA [23,24,25,26,27,28,29], this study aimed to examine the effects of Personalized Observation, Execution, and Mental Imagery (POEM) therapy on verb retrieval in lvPPA within a telepractice context. POEM was created based on the observation that language and motor systems interact, in accordance with the principles of embodied cognition [30]. According to this theoretical framework, cognitive processes are grounded in bodily states and sensorimotor experiences, such that conceptual knowledge is shaped and supported by perceptual and action systems [31]. Building on this interaction, Durand et al. [30,32] exploited the synergy between motor and linguistic processes, as well as the underlying mechanisms that support the effectiveness of such approaches to aid in the rehabilitation of people with chronic post-stroke aphasia. In particular, POEM, which integrates three sensorimotor strategies to facilitate action verb naming—observation of action videos, motor execution, and mental imagery—has been shown to yield significant gains in verb retrieval in persons with chronic post-stroke aphasia: action naming improved significantly, with evidence of generalization effects [30]. Importantly, gains were maintained over time, up to six months post-therapy at the final protocol retest. These behavioral improvements were accompanied by increased activation in both language-related and sensorimotor brain regions, further supporting the hypothesis that their functional interplay contributes to language recovery [30,32]. In summary, POEM therapy is evidence-based and incorporates targeted, repetitive, and intensive action-naming practice to promote strategy acquisition and integration. While it is traditionally delivered face-to-face by a speech and language therapist (SLT) for individuals with chronic vascular aphasia, it may also be beneficial for persons with degenerative aphasia, either in person or via telepractice.

Telepractice is increasingly considered a valid technique for the management of neurodegenerative disorders, including PPA. Indeed, studies have demonstrated the clinical feasibility of telepractice for individuals with PPA and related populations, supporting its use as an effective alternative to face-to-face intervention [33,34,35]. Telepractice may help address challenges related to reduced healthcare costs, but also reduced mobility, disease progression, and regional disparities in access to specialized speech and language therapy services. Telepractice also provides the advantage of improving the frequency and continuity of care [36].

In line with the aim of this study, we hypothesized that naming accuracy for both trained and untrained verbs would improve following the intervention. In addition, we expected improvements in functional communication and connected speech.

## 2. Materials and Methods

### 2.1. Participant

The participant, assigned the pseudonym “Marie,” was a 74-year-old right-handed native French speaker. Marie was recruited for the study once she met the inclusion and exclusion criteria, namely being a native French speaker and having a diagnosis of lvPPA [3]. She was married, had two children, and had completed nine years of formal education. She had previously worked as a housekeeper and was retired at the time of the study. She had been diagnosed with lvPPA six months prior to inclusion. The diagnosis was established on the basis of clinical examination, neuropsychological assessment, and structural MRI, in accordance with the consensus criteria proposed by Gorno-Tempini et al. [3]. Her clinical presentation was consistent with lvPPA. She reported progressive word-finding difficulties and described frequent tip-of-the-tongue states, leading to a marked reduction in spontaneous verbal communication, including with close family members. Because of these lexical retrieval difficulties, she avoided answering telephone calls. Although she indicated that her social life had never been particularly extensive, she reported increasing social withdrawal over time, a change confirmed by her husband. She also endorsed mild depressive symptoms, characterized by persistent sadness. These clinical features, including depressive symptoms and reduced social engagement, may have influenced both participation in the intervention and variability in performance across sessions. At the time of the study, she was receiving speech and language therapy three times per week. Psychological support had recently been initiated in response to her depressive symptoms.

### 2.2. Study Design

This study was designed as a single-case experimental design (SCED) [37,38,39]. Marie served as her own control (intrasubject analysis), and her performance was assessed repeatedly on the same items at frequent intervals throughout the protocol (repeated measures). However, we were only able to recruit one person with lvPPA. We chose to keep the repeated measures, as this design is considered stronger than a pre- and post-test design, but the sequential introduction of the intervention (randomization of the baseline’s length) could not be implemented due to the inclusion of a single participant. Although baseline length could not be randomized because it had only one participant, the use of repeated measures strengthens internal validity by allowing for continuous monitoring of performance over time. The study followed an ABA design: the initial phase A corresponded to the three administrations of the 121 baseline verbs conducted prior to therapy; phase B corresponded to POEM administration following a previously tested dosage [30,32] in three weekly sessions lasting 45 min each, over a five-week period, resulting in a total of 15 scheduled sessions with the participant; and the final phase A corresponded to the post-therapy assessment phase, which used the same tests as in the initial phase A.

### 2.3. Pre- and Post-POEM Assessments

Two assessments were carried out: the first prior to the intervention, aimed at establishing a comprehensive cognitive profile of Marie, and the second at the end of the therapeutic intervention, intended to objectively evaluate the changes in Marie’s performance (Table 1).

Questionnaires and tasks were administered to assess Marie’s functioning across multiple domains. The pre- and post-therapy assessments indicated stable performance across most linguistic domains, with modest improvements observed in gesture production and selected aspects of functional communication (Table 1).

For the analysis of narrative discourse in the Cinderella task, each narrative production of Cinderella was recorded on video in the pre-therapeutic and post-therapeutic phases. The recordings were made in accordance with the methodology described by Stark et al. [52].

### 2.4. POEM Therapy Material

The POEM material consisted of 121 video stimuli developed by ED during her doctoral thesis under the supervision of Ana Inés Ansaldo. Each 5 s video depicts an older man or woman performing an action in an ecologically congruent environment (e.g., swimming in a pool, watering plants in a garden) (see [30] for details).

### 2.5. POEM Therapy

**Figure 1:**
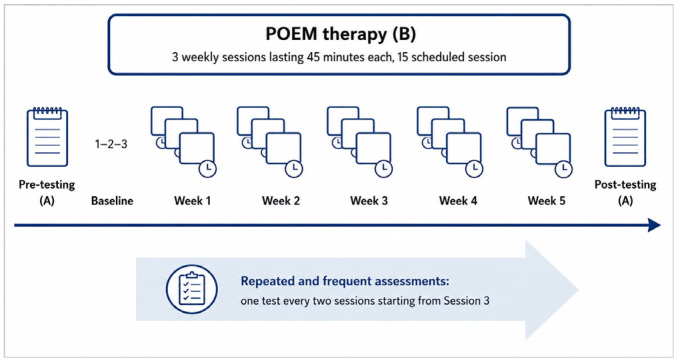
Experimental design.

Figure 1 shows the experimental design.

#### 2.5.1. Development of Trained and Untrained Verb Lists

POEM therapy was delivered across fifteen 45 min sessions, three times per week over a five-week period. Baseline performance was assessed three times prior to intervention using a video-based action-naming task comprising the 121 verbs, which resulted in the identification of 16 consistently failed items across the three assessments, plus four that were successfully produced in the three naming trials to reinforce motivation and verify the prophylactic effect, i.e., the maintenance of the preserved elements. In addition, a set of 40 untrained items was constructed, matched on frequency, number of syllables, and valence (*p* > 0.05). The full list of trained and untrained items is provided in Appendix A. The therapist (HS) received prior training in the POEM protocol from an experienced SLT (ED).

#### 2.5.2. Within-Protocol Repeated Measures

Marie’s performance was repeatedly assessed at a rate of one probe every two sessions, beginning with the third session, in order to evaluate the effects of the intervention on verb retrieval. The probe list comprised six verbs selected from the trained set based on frequency (high vs. low), length (short vs. long), and argument structure (monovalent vs. bivalent).

During the study period, Marie did not receive any speech–language therapy other than the POEM protocol. As noted above, she attended three speech–language therapy sessions per week, all of which were exclusively devoted to POEM. This helped minimize potential confounding effects from other therapeutic approaches and facilitated the interpretation of treatment-related changes.

### 2.6. Procedure

The intervention was conducted remotely due to geographic constraints, using the Microsoft Teams platform^®^. This platform was selected for its user-friendly interface and ease of scheduling, which facilitated its use by both the participant and the investigator and ensured smooth session delivery. In addition, the platform ensured the confidentiality of exchanges and did not involve the recording or storage of session content.

To minimize potential bias, the SLT working with Marie was instructed not to provide any verbal or non-verbal facilitation during the telerehabilitation sessions and was limited to a supervisory role.

Marie was seated upright with her arms free and viewed the stimuli on a computer screen. Each video was presented using PowerPoint, with a white screen between trials.

All sessions followed a standardized timing, item presentation, and prompting procedures to ensure consistency across the intervention period. During each session, the 20 trained items were presented in a randomized order. Each item was administered according to a three-step hierarchical procedure: (1) video presentation (e.g., to wash) with the prompt “What is this person doing?” to elicit action naming in the infinitive form; (2) if the participant did not respond within 10 s, Marie was asked to produce the corresponding gesture, with assistance if required, following the Instruction “Show me how this person does it with your hands”; (3) if the gesture was unsuccessful, Marie was instructed to engage in mental imagery of the action in a personal context (e.g., “Close your eyes and imagine this action in your kitchen”). After each step, the target word was provided and repeated once before proceeding to the next item.

Once all 20 items had been administered, the full procedure was repeated a second time in the same session.

Every other session from session 3 onward, the six verbs selected to constitute the repeated measures were presented to Marie in the form of a slideshow similar to that used in the protocol. HS asked Marie to name the verb; however, if she failed to do so, no prompt was offered, and the next verb was presented.

Naming responses were scored and sensorimotor strategies were coded jointly by HS and the SLT present during the therapy sessions.

### 2.7. Analyses

Treatment outcomes were evaluated using measures targeting lexical retrieval, functional communication, and connected speech. The primary outcome measure was verb-naming accuracy for trained items, reflecting the main objective of the intervention. Naming accuracy was calculated as the proportion of correctly produced verbs during repeated naming probes administered across study phases. To evaluate the effects of the sensorimotor strategies, two outcome measures were considered: (1) the number of correctly produced verbs during the initial spontaneous naming phase, and (2) the total number of correctly produced verbs across the three phases of the protocol. Scoring of action naming took into account both (1) production performance during the presentation of the action video, scored as 1 for a correct response (target verb produced) and 0 if the target was not produced; and (2) when the target was not produced, the speech–language therapist (HS) recorded the sensorimotor strategies used to elicit the target, again scoring 1 for a correct response (target verb produced) and 0 if the target was not produced.

To assess generalization, naming accuracy for untrained verbs was also examined. This measure allowed us to determine whether treatment gains extended beyond the trained lexical items. Secondary outcome measures included indices of functional communication, obtained with a functional communication questionnaire administered before and after the intervention, as well as measures of connected speech derived from discourse samples. Connected speech analyses focused on indicators of verb production and overall informativeness.

Analyses were conducted in accordance with SCED guidelines [53,54], combining visual and statistical approaches to evaluate intervention effects. Visual analysis, including inspection of level, trend, and variability, remains the primary method in SCED but is increasingly complemented by statistical indices to enhance interpretability and rigor [37]. This combined approach is consistent with current recommendations in the aphasia and rehabilitation literature [55,56,57].

#### 2.7.1. Visual Analysis

Visual analyses were supported by dedicated online tools: http://ktarlow.com/stats/tau/ (accessed on 20 April 2025); https://jepusto.shinyapps.io/SCD-effect-sizes/ (accessed on 20 April 2025).

Baseline trend stability was first examined to distinguish treatment-specific effects from potential spontaneous recovery, that is, to determine whether any changes observed during the intervention could be attributed to treatment rather than to spontaneous recovery or pre-existing performance trends. The dual-criterion (DC) method was then applied to assess intervention effects, with data points exceeding the projected trend line interpreted as performance above baseline expectations, thereby providing evidence of treatment-related change. In addition, the two-standard-deviation band (2-SDB) method was used to define a ±2 SD envelope around the baseline mean to identify changes that exceeded normal baseline variability. A treatment effect was considered to exist when at least two consecutive data points during the intervention phase exceeded this band [37].

#### 2.7.2. Statistical Analysis

Statistical analyses included the Non-overlap of All Pairs (NAP), which quantifies the proportion of non-overlapping data points between the baseline (Phase A) and intervention (Phase B). NAP was used to estimate the magnitude of the treatment effect. Higher NAP values indicate stronger treatment effects and are particularly informative in the presence of variability. According to established benchmarks, values of 0–31% indicate a weak or negligible effect, 32–84% a moderate effect, and ≥85% a strong effect [58]. The Baseline-Corrected Tau (BC-Tau; [59]) was also computed to account for baseline trends and provide an effect size estimate with an associated *p*-value [55,56,57]. This analysis was applied to determine whether observed changes remained significant after controlling for baseline trends.

In addition, a Wilcoxon signed-rank test was conducted to compare performance on trained verbs between pre- and post-therapy phases, as well as to assess changes in performance for both trained and untrained verbs. This test assessed whether performance improved following the intervention. A Friedman test was performed to examine the presence of a practice effect across the three administrations of the action verb video set. Finally, performance on pre- and post-assessment tasks was analyzed using raw scores and compared across administrations. This analysis was used to determine whether repeated exposure to the assessment materials influenced performance.

#### 2.7.3. Functional Communication and Connected Speech Analysis

For the analysis of narrative discourse in the Cinderella task, speech samples were transcribed and analyzed using CLAN (Computerized Language ANalysis; version 28-sept-2023), a component of the TalkBank system [43], following the CHAT (TalkBank) methodology. Only Marie’s utterances were analyzed. Two automatic analyses were carried out in CLAN: (1) EVAL, which generates a summary of the linguistic characteristics of a speech sample; and (2) FREQ, which calculates the frequency of occurrence of lexical units. The analysis of Marie’s Cinderella narratives is based exclusively on a descriptive approach.

## 3. Results

### 3.1. Effects of POEM Therapy on Trained Verbs

#### 3.1.1. Visual Statistical Analysis

##### Baseline Trend Stability

**Figure 2:**
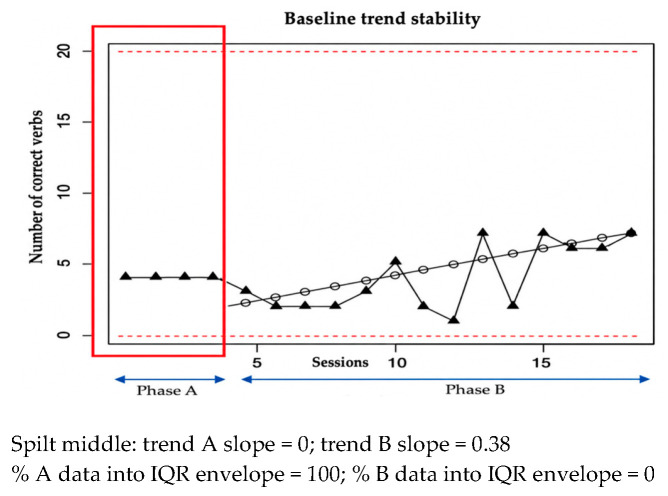
Visual analysis of baseline trend stability. *Note*. Red dashed lines = interquartile range (Q1–Q3) of Phase A; IQR = interquartile range.

During the baseline phase (Phase A), performance remained stable. As shown in Figure 2, all data points fell within the baseline envelope, with no observable upward trend in naming accuracy prior to the intervention. Marie consistently produced 4/20 correct responses across all three baseline assessments, resulting in a standard deviation of 0. These results indicate a stable baseline with no evidence of spontaneous improvement prior to therapy.

In summary, the stability of baseline performance may suggest that any subsequent changes are unlikely to reflect spontaneous recovery.

##### Dual-Criterion Analysis

**Figure 3:**
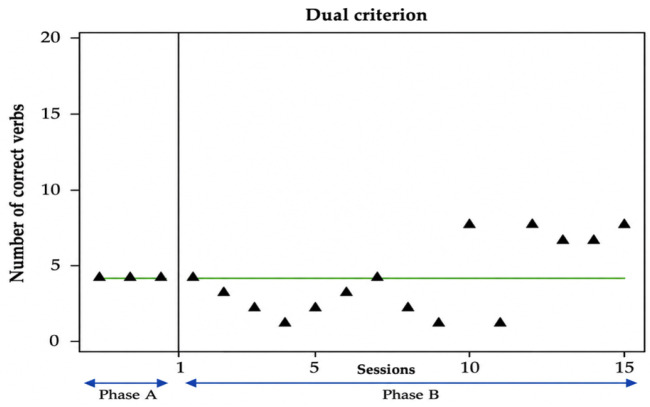
Visual analysis of POEM treatment outcomes across the naming task, based on the Dual-Criterion Test. *Note*. Green line: trend line (mean level of Phase A extended into Phase B).

**Figure 4:**
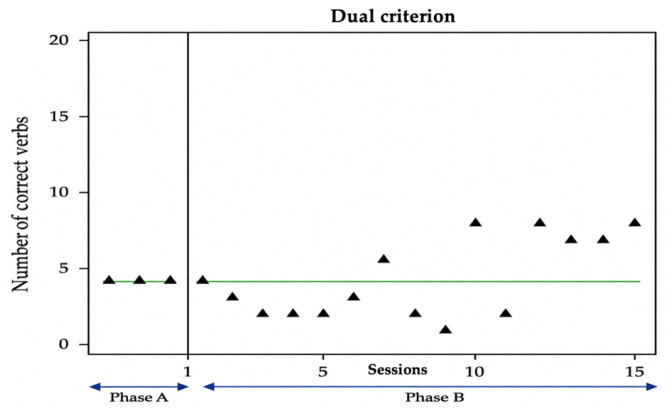
Visual analysis of naming performance across the three POEM strategies, based on the Dual-Criterion Test. *Note*. Green line: trend line (mean level of Phase A extended into Phase B).

The DC method was applied to evaluate potential treatment effects. As Figure 3 and Figure 4 show, several intervention data points exceeded the projected criterion line (green), suggesting a change in performance relative to baseline expectations.

Taken together, these findings may indicate changes in performance relative to baseline levels. However, given the lack of baseline variability and trend, the results should be interpreted cautiously and considered only as preliminary indications of a possible treatment-related effect.

##### Intervention Phase

**Figure 5:**
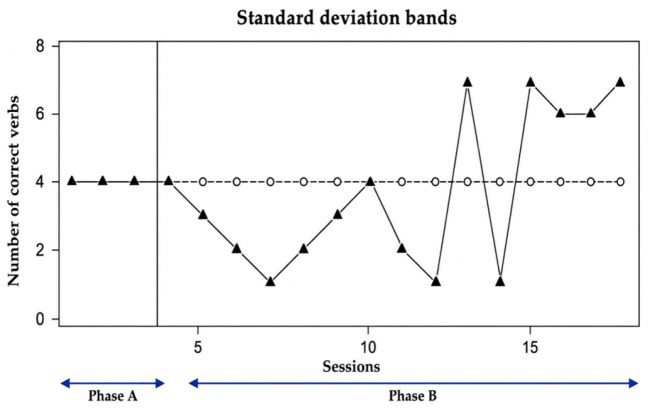
Visual analysis of POEM treatment effects during the naming task using the 2-SDB method.

**Figure 6:**
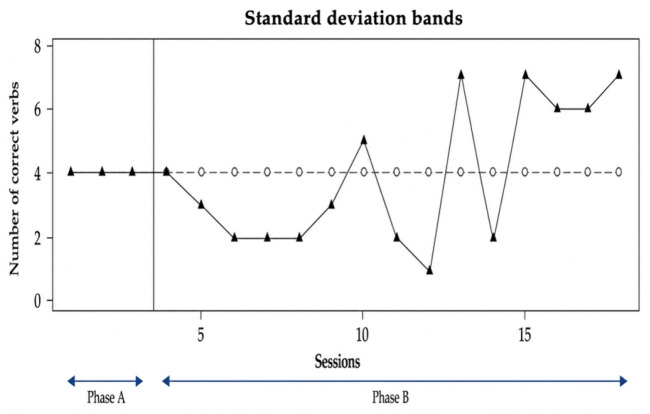
Visual analysis of POEM treatment effects across the three strategies using the 2-SDB method.

During the intervention phase (Phase B), Marie’s naming performance showed increased variability across sessions (Figure 5 and Figure 6). Scores mostly remained below the mean until session 9. From session 10 onward, performance increased and stabilized at around 6 or 7 correctly named verbs per session. Visual inspection revealed an overall upward trend in naming accuracy across the intervention phase.

For 2-SDB analysis, baseline scores showed no variability (SD = 0), resulting in an overlap of the mean and trend lines and preventing visualization of the 2-SD band. Consequently, any score above the baseline exceeded the 2-SD criterion. As shown in Figure 5 and Figure 6, four consecutive data points during the final therapy sessions exceeded this threshold.

Collectively, these findings may suggest a gradual improvement in naming performance during the later stages of the intervention. However, because baseline scores showed no variability and were based on a limited number of observations, the resulting 2-SDB criterion should be interpreted with caution, as the lack of baseline variance may not fully capture natural performance fluctuations and could overestimate the apparent significance of observed changes.

#### 3.1.2. Statistical Analysis

**Figure 7:**
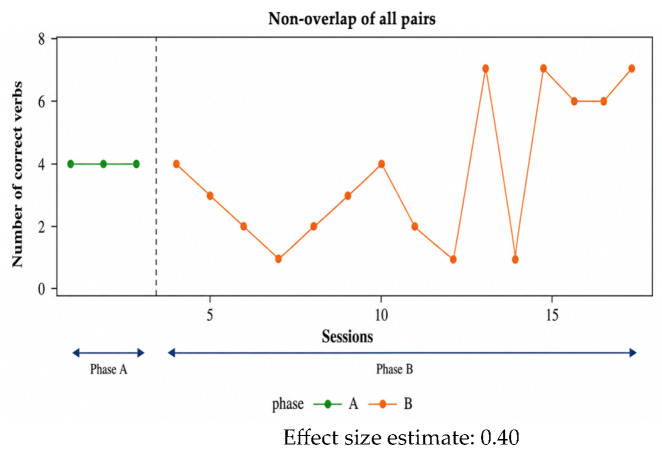
Visual analysis of POEM treatment effects during the naming task using NAP.

**Figure 8:**
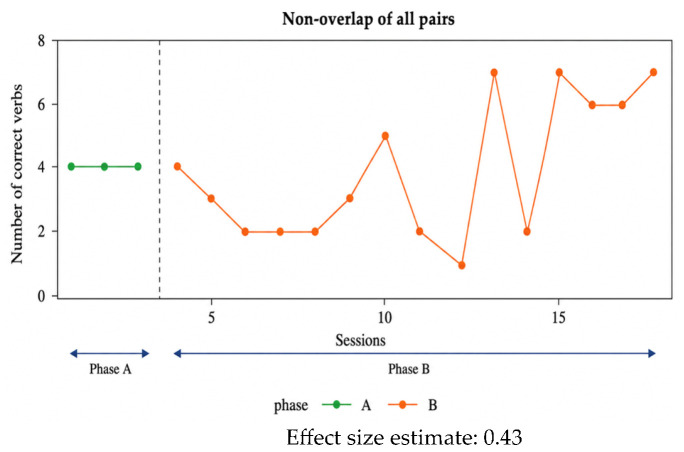
Visual analysis of POEM treatment effects across the three strategies using NAP.

The NAP analysis indicated a moderate treatment effect. NAP values were 40% for the naming task (Figure 7) and 43% when all three strategies—observation, gesture, and mental imagery (Figure 8)—were considered.

To account for potential baseline trends, a BC-Tau analysis was conducted. The comparison between phases A and B was not statistically significant (three strategies: Tau = −0.078, *p* = 0.763; naming alone: Tau = −0.117, *p* = 0.632), indicating a small treatment effect. No trend correction was required, as baseline performance showed no variability.

A Wilcoxon signed-rank test comparing pre- and post-therapy performance on trained verbs revealed no significant difference (W = 232.0, *p* = 1.00), indicating that overall naming performance remained stable across assessments.

Collectively, these findings indicate limited quantitative evidence of a treatment effect. Although some analyses suggested changes in performance during the intervention phase, the BC-Tau analysis was not statistically significant. NAP values were modest, and no significant gains were observed on the pre–post naming assessment. Consequently, the results should be interpreted cautiously and do not provide strong evidence for improvements in verb-naming performance following the intervention.

### 3.2. Effects of POEM Therapy on Naming Performance on Untrained Verbs

#### 3.2.1. Statistical Analyses

A Friedman test was conducted to examine the potential effects of repeated exposure across assessments and showed no significant differences (χ^2^ = 0.150, *p* = 0.92), suggesting that there was no practice effect. Consequently, Wilcoxon signed-rank tests were used to further assess the effect of therapy on naming performance for trained and untrained verbs. Comparisons between mean pre-treatment performance across the three baseline assessments and post-treatment performance showed no statistically significant differences (Baseline 1: W = 232.0, *p* = 0.45; Baseline 2: W = 110.0, *p* = 0.50; Baseline 3: W = 232.0, *p* = 0.23). Overall, these results indicate that naming performance did not change significantly following therapy.

Although these results did not achieve statistical significance, the stabilization of performance may still be considered clinically meaningful in the context of a progressive neurodegenerative condition. However, these results should be considered with caution, given the potential influence of unmodeled temporal dependencies between repeated measurements.

#### 3.2.2. Qualitative Results: Co-Verbal Gestures During Naming Task

Analyses revealed an increase in the use of action gestures as a facilitative strategy. At the post-therapy assessment, a correctly executed action gesture preceded a verbal attempt in 19 out of 25 occurrences, representing 76% of verbal attempts accompanied by gesture production.

To quantify this change, Wilcoxon signed-rank tests were conducted to compare gesture use across the three pre-therapy assessments and the post-therapy assessment. Comparisons with the second and third pre-therapy assessments did not reach statistical significance (administration 2, *p* = 0.147; administration 3, *p* = 0.262). In contrast, the difference between the first pre-therapy administration and the post-therapy assessment was statistically significant (*p* < 0.001).

### 3.3. Effects of POEM on Functional Communication

Table 1 presents the results Marie obtained in various domains before and after therapy.

Functional communication was assessed using the CETI [42], which was completed independently by Marie, her husband, and the SLT to capture everyday communication from multiple perspectives.

Pre-therapy, a marked discrepancy was observed between Marie’s self-ratings (mean = 6.13/10) and those provided by the SLT (3.44/10) and her husband (4.38/10). For conversational items (7 items), Marie reported relatively high functioning (mean = 7/10), whereas the SLT (1.43/10) and her husband (3.14/10) reported that she had substantial difficulties.

Post-therapy, the discrepancy between raters persisted. Mean scores increased slightly for Marie (6.56/10) and her husband (5.50/10). Improvements were observed in several ecologically relevant items, including attracting attention, participating in multi-speaker conversations, and elaborating on a topic. Conversational items also received higher scores across raters, particularly for the husband (5.29/10 vs. 3.14/10 pre-therapy).

A one-tailed Wilcoxon signed-rank test (H1 > H0) comparing pre- and post-therapy scores revealed no significant changes for Marie (W = 1.00, *p* = 0.21) or the SLT (W = 0.00, *p* = 0.50). However, her husband’s ratings of her performance improved significantly (W = 0.00, *p* = 0.02).

Overall, these findings suggest that Marie’s functional communication improved to some extent following therapy, reflected primarily in her husband’s ratings.

### 3.4. Effects of Therapy on Connected Speech

In the Cinderella story retelling task, Marie exhibited marked anxiety in the pre-therapy condition. She said she found it difficult to complete the task and remained largely silent, producing no verbs. This profile is consistent with previous reports of reduced verb production in the connected speech of individuals with aphasia (e.g., ref. [24]).

Post-therapy, although some apprehension persisted, Marie was able to engage in the narrative task. She identified the main characters, maintained the chronological sequence of events, and produced short but informative utterances (e.g., “the prince is looking for someone”; “the two girls are planning something”; “at the end, they kiss”).

A linguistic analysis of connected speech was conducted using EVAL (CLAN). As Table 2 shows, several indicators suggested increased discourse production following therapy. Narrative duration increased substantially (76 s vs. 253 s), and the number of utterances nearly doubled (15 vs. 29). Mean length of utterance in terms of both words and morphemes also increased. Lexical diversity (types and tokens) and the proportion of several grammatical categories, including prepositions, conjunctions, determiners, and pronouns, increased, suggesting greater syntactic elaboration in the post-therapy condition.

Unexpectedly, the EVAL output indicated a decrease in the proportion of verbs. Visual inspection of the transcripts suggested that this result was influenced by meta-comments produced outside the narrative (e.g., “I don’t know”), which were included in the EVAL calculations. To clarify this pattern, a complementary FREQ analysis was conducted, focusing specifically on verbs produced within the narrative.

The FREQ analysis distinguished verb tokens (total occurrences) from verb types (different verbs). Following the classification proposed by [60], verbs were also categorized as light verbs (low semantic specificity, e.g., go, do) and heavy verbs (higher semantic specificity, e.g., find, search).

In the pre-therapy condition, 12 verb tokens and 7 verb types were identified, of which only three occurred within the narrative. Production was dominated by light verbs. In contrast, the post-therapy condition showed 35 verb tokens and 21 verb types, with 11 produced within the narrative itself, representing an approximately threefold increase. In addition to several light verbs, Marie produced more specific heavy verbs (e.g., close, calculate, kiss), contributing to greater narrative precision.

Overall, the FREQ analysis indicated an increase in both the quantity and semantic specificity of the verbs Marie produced within the narrative following POEM therapy.

Table 2 shows the results for Marie’s narrative discourse.

These findings suggest that POEM therapy may support improvements in discourse-level language production, which is particularly relevant for everyday communication.

## 4. Discussion

This study investigated the effects of POEM therapy on Marie, a person with lvPPA presenting with verb anomia. A comprehensive neuropsychological assessment was conducted before and after the intervention. Marie completed 15 POEM therapy sessions, delivered three times per week via telepractice due to geographic constraints. We hypothesized that the therapy would result in improved naming of both trained and untrained verbs. In addition, we expected gains in functional communication, as measured by a functional communication questionnaire, and in narrative discourse.

### 4.1. Effects of POEM on Trained Items

We remind readers that the SCED methodology could not be fully implemented in this study, as randomization of baseline length was not possible with a single participant. Consequently, the findings should be interpreted with appropriate caution, not as evidence from a fully controlled SCED. Thus, the design provides moderate support for treatment-related changes. The statistical analyses revealed no significant improvement in naming accuracy for trained verbs following POEM therapy. However, Marie’s performance was maintained, indicating that her abilities had stabilized, which is clinically meaningful. As reported in the literature, PPA is a neurodegenerative condition characterized by progressive language decline [3,61]. In this context, therapeutic interventions primarily aim to preserve retained skills rather than to restore language functions, in contrast to rehabilitation approaches for post-stroke aphasia [35].

Several factors may account for the lack of a significant improvement. Executive dysfunction, which is frequently described in PPA, may have limited the systematic application and automation of the strategies required during video-based training. CASP results confirmed the existence of executive difficulties likely to interfere with protocol adherence [14,62]. Moreover, POEM relies on motor execution and mental imagery strategies; baseline assessments revealed praxis impairments and deficits in motor imagery, potentially complicating the integration of these novel strategies in a context of neurodegeneration.

Marie’s mood disturbances may also have contributed to the observed outcomes. Anxiety and depressive symptoms are commonly reported in PPA [63,64]. During the study, clinically significant anxiety followed by a diagnosis of depression may have contributed to Marie’s variable performance, repeated pauses, and occasional session interruptions, thereby reducing the actual intensity of training despite verb randomization.

Nevertheless, visual analyses demonstrated a significant improvement during the last four sessions, suggesting a delayed therapeutic effect. This delayed effect may reflect the time required to internalize compensatory strategies in a neurodegenerative disorder, particularly when executive deficits exist [14]. Increased spontaneous use of gestures during communication and naming tasks may also suggest the progressive integration of the motor-based strategy promoted by POEM.

Overall, although no immediate significant gains were observed, the delayed positive trend suggests that administering POEM for a longer time may yield more robust and measurable effects.

### 4.2. Effects of POEM on Untrained Items

No significant improvement was demonstrated in naming accuracy for untrained verbs following POEM, indicating a lack of generalization to untreated items. This finding does not corroborate previous studies suggesting that generalization effects may be more prevalent in lvPPA [65,66]. Marie’s executive, neurocognitive, and mood problems, as well as the duration of the intervention, may account for the lack of transfer. Nevertheless, statistical analyses showed that items and lexical concepts preserved prior to therapy remained stable post-intervention, supporting a prophylactic effect consistent with maintenance-oriented approaches in neurodegenerative conditions [67]. These findings may suggest that, although lexical generalization was not observed, strategic generalization occurred, which was reflected in the increased use of motor-based compensatory mechanisms during verb retrieval.

### 4.3. Effects of POEM on Functional Communication

Marie’s functional communication scores did not increase significantly following the administration of POEM. However, analyses revealed a significant change in the ratings provided by her husband, suggesting that he perceived improvements in her everyday communication. As the participant’s primary communication partner, his observations may provide valuable insight into potential changes occurring in daily interactions. Nevertheless, these caregiver-reported outcomes should be interpreted with caution, as they are subjective and may be influenced by unmeasured confounding factors. These results are in line with recent studies showing that interventions are required to improve functional communication in real-world contexts [68].

Moreover, qualitative analysis of Marie’s self-ratings revealed changes between pre- and post-therapy testing. She reported feeling more able to attract someone’s attention, indicate that she understands what is being said to her, or take part in a quick conversation with several people. These elements may suggest that Marie feels more invested in communication and wants to take part in conversations.

### 4.4. Effects of POEM on Connected Speech

In the pre-therapy condition, Marie produced no verbs. This profile is consistent with previous reports of reduced verb production in spontaneous speech in lvPPA [24]. Marie’s results showed an improvement in the number of verbs she produced during narrative speech after the therapy. This finding is particularly encouraging given the severe anomia typically observed in people with lvPPA [28,61]. The post-therapy increase in verb production during the Cinderella narrative suggests a potential beneficial effect of the intervention on verb retrieval in narrative speech. These findings are consistent with previous studies demonstrating improved lexical retrieval following interventions targeting that skill in connected speech in individuals with PPA [69,70,71]. However, this interpretation should be made with caution, as a possible test–retest or familiarity effect cannot be ruled out.

### 4.5. Overview of Results

In summary, this study aimed to examine the effects of POEM therapy on a person with lvPPA in the context of a speech and language therapy telepractice. The results suggest that a therapeutic impact was made at several levels of communication. In line with guidelines for intervention in neurodegenerative disorders, the main objective was to maintain abilities. In this study, POEM specifically targeted verb anomia. Analyses revealed that verb-naming performance stabilized, indicating an initial therapeutic effect.

While the level of competence in verb-naming remained stable, performance in narrative discourse improved: indeed, FREQ analysis confirmed an increase in verb production in discourse situations. Although narrative speech is a semi-structured task, it is similar to spontaneous speech and can therefore provide information about the potential functional impact on everyday communication. These results support the hypothesis that POEM could have a positive effect on verb production in connected speech.

Functional communication measures also provided clinically meaningful data. While Marie’s self-report did not show significant improvement, the score her husband gave her increased significantly, indicating a perceived enhancement in daily communicative interactions. This finding is particularly relevant, as the purpose of speech and language therapy focused on language is to enable people with PPA and their carers to communicate more fluently, effectively and pleasantly, resulting in a healthier daily life [65,68].

From a theoretical point of view, these results are consistent with the conceptual frameworks of embodied and situated cognition, which postulate that language—particularly action-related verbs—is based on distributed sensorimotor networks. Neurocognitive models suggest that verb processing involves the activation of the motor and premotor cortices, reflecting the simulation of action representations. From this perspective, gestures can act as externalized motor signals that reinforce or reactivate partially degraded lexical–semantic networks by engaging preserved sensorimotor circuits. In a situated cognition framework, meaning emerges through the dynamic interaction between the individual, the body, and the environment; thus, incorporating gestures into therapy can facilitate lexical access by embedding language in action-oriented contexts. The improvements observed in narrative discourse also suggest a functional impact on continuous discourse, which is closer to everyday communication situations.

Overall, while immediate improvements in naming accuracy were not observed, the stabilization of performance, combined with delayed gains in later sessions, suggests that POEM may contribute to both maintenance and gradual improvement over time. These findings support the hypothesis that POEM has beneficial effects on a person with lvPPA within the context of telepractice speech and language therapy, with improvements observed at the linguistic, discourse, and functional communication levels.

Although the results are promising, this study presents several limitations. The SCED methodology could not be fully implemented: indeed, as the study involved only a single participant, randomization was not possible. A follow-up assessment to evaluate the maintenance of performance one month after the end of therapy could not be conducted due to time constraints. The statistical findings should be interpreted with caution. The limited number of baseline observations resulted in no baseline variability, which may have affected the sensitivity and interpretability of the 2-SD Band and dual-criterion analyses. In addition, the modest NAP values, non-significant BC-Tau results, and absence of explicit modeling of autocorrelation between repeated measurements limit the strength of inferences regarding treatment effects. Future studies with larger samples and longer observation periods should incorporate statistical approaches that account for within-subject variability and temporal dependence.

From a clinical perspective, these findings tentatively support the incorporation of gesture-based approaches in verb-oriented aphasia therapy. Encouraging the production of semantically congruent gestures may help recruit preserved sensorimotor circuits and promote compensatory pathways for lexical access.

## 5. Conclusions

To sum up, this study was the first to investigate the effects of POEM therapy on a participant with lvPPA within the context of remote speech and language therapy.

This study revealed no statistically significant differences between pre- and post-therapy performance on trained and untrained verbs. However, qualitative analyses suggest that the participant demonstrated emerging improvements. In particular, gains were observed in verb initiation and partial word production. These results may reflect subtle changes in lexical access processes potentially related to the POEM therapy, which were not captured by quantitative measures. Taken together, these findings provide limited preliminary evidence regarding the effects of POEM on verb production in lvPPA. Nevertheless, they indicate that the intervention is feasible in this population and may support compensatory strategies, including gesture use, particularly in telepractice contexts. Overall, while the results do not allow firm conclusions about efficacy, they suggest that POEM may be a clinically relevant sensorimotor-based approach to compensate for the underlying linguistic deficits in people with lvPPA.

POEM provided promising results in this case, supporting the clinical relevance of sensorimotor interventions for treating verbs in lvPPA. POEM may therefore promote compensatory neurocognitive mechanisms through sensorimotor engagement. Future research involving larger samples and longer intervention periods, using standardized and adapted administration protocols, is needed to determine whether gesture-mediated facilitation produces robust, generalizable effects. Future studies should also directly compare in-person and remote delivery formats to determine the generalizability, feasibility, and strength of POEM therapy across clinical settings.

## Figures and Tables

**Table 1:** Pre- and post-POEM assessment results.

	Pre-POEM Assessment	Post-POEM Assessment	Cut-Off Score
**LINGUISTIC FUNCTIONS**			
Fluency—Fruits	2	1	18.5
Fluency—Verbs	0	0	17
Fluency—Letters	2	0	12.5
Words Repetition	7 out of 10	8 out of 10	10 out of 10
Nonword repetition	3 out of 6	3 out of 6	6 out of 6
- Nonwords	2 out of 3	2 out of 3	3 out of 3
- Pseudowords	1 out of 3	1 out of 3	3 out of 3
Sentence repetition	13 out of 30	15 out of 30	30 out of 30
- Long sentences	10 out of 12	7 out of 12	12 out of 12
- Short sentences	3 out of 18	7 out of 18	18 out of 18
Syntactic comprehension	10 out of 24	9 out of 24	22 out of 24
DVAQ-30	1 out of 30	2 out of 30	24 out of 30
CETI	Marie: 6.13 SLT: 3.44 Spouse: 4.38	Marie: 6.56 SLT: 3.38 Spouse: 5.50	
Cinderella speech task	See Section 3.4.	See Section 3.4.	
**COGNITIVE FUNCTIONS**			
TMT (>60 years old, level 1)			
- Mistakes	<3.8	<3.8	0
- Time	<202 s	<202 s	90 s
RFFT			
- Part I	4	6	17.3
- Part II	4	4	18.9
- Part III	5	4	19.1
CASP	23 out of 36	25.5 out of 36	35 out of 36
**MOTOR ABILITIES**			
Mahieux Praxis	13 out of 23	16 out of 23	18 out of 23
- Symbolic gestures	3 out of 5	2 out of 5	4 out of 5
- Pantomimes	5 out of 10	8 out of 10	8 out of 10
- Imitation of meaningless gestures	5 out of 8	6 out of 8	6 out of 8
KVIQ-10	10	27	50–55
Paris Praxis	4 out of 4	4 out of 4	4 out of 4
**MOOD**			
GAI	13 out of 20	8 out of 20	≤8 out of 20
GDS	Partially completed	6	5

*Note.* Linguistic functions: GréMots (verbal fluency; word, nonword and sentence repetition, syntactic comprehension) [40]; DVAQ-30 [41]; CETI, Communicative Effectiveness Index [42]; Cinderella speech task [43]. Cognitive functions: TMT, Trail Making Test [44]; RFFT, Ruff Figural Fluency Test [45]; CASP, Cognitive Assessment Scale for Stroke Patients [46]. Motor abilities: Mahieux test [47]; KVIQ-10, Kinesthetic and Visual Imagery Questionnaire—10 items [48]; bucco-facial praxis (PARIS protocol) [49]. Mood: GAI, Geriatric Anxiety Inventory [50]; GDS, Geriatric Depression Scale [51].

**Table 2:** CLAN EVAL results for narrative discourse: pre- vs. post-therapy.

	Pre-Therapy	Post-Therapy
Duration of linguistic content (s)	76	253
Total utterances	15	29
Mean length of utterances (words)	15	28
Mean length of utterances (morphemes)	4.867	9.071
Number of different words	5.8	10.107
Total number of words produced	41	94
Mean words per minute	108.158	111.462
Verbs per utterance	0.8	0.655
Percentage of nouns in the corpus	16.092	19.259
Percentage of correct plurals in the corpus	28.571	32.692
Percentage of verbs in the corpus	13.793	7.037
Percentage of prepositions in the corpus	4.598	8.519
Percentage of adjectives in the corpus	1.149	2.963
Percentage of adverbs in the corpus	14.943	15.185
Percentage of conjunctions in the corpus	8.046	11.481
Percentage of determiners in the corpus	4.598	9.63
Percentage of pronouns in the corpus	36.782	40.37

*Note*. s: seconds.

## Data Availability

The data are not publicly available due to privacy and ethical restrictions.

## Appendix A

**Table A1:** List of Trained Verbs.

VERB	FREQUENCY-FILMS	NUMBERPHONEMES	CV	NUMBERLETTERS	VALENCE
Laver (to clean)	34.01	4	CVCVC	5	P0 (P1)
Acheter (to buy)	115.27	5	VCCVCVC	7	P0 P1 (P3)
Verser (to pour)	4.62	5	CVCCVC	6	P0 P1 (PP<in>)
Effacer (to erase)	10.05	5	VCCVCVC	7	P0 P1
Écraser (to mash)	16.75	6	VCCVCVC	7	P0 P1 (PP<on>)
Repasser (to iron)	7.27	6	CVCVCCVC	8	P0 (PL) (PT)
Chuchoter (to whisper)	1.46	6	CCVCCVCVC	9	P0 (P1) (P2)
Débrancher (to unplug)	2.27	7	CVCCVCCCVC	10	P0 P1
Applaudir (to applause)	3.16	7	VCCCVVCVC	9	P0
Scier (to saw)	1.12	3	CCVVC	5	P0 (P1)
Compter (to count)	45.05	4	CVCCCVC	7	P0 P1 (PP<to>)
Peindre (to paint)	12.75	4	CVVCCCV	7	P0 (P1) (PM)
Peler (to peel)	0.44	4	CVCVC	5	P0 P1
Sentir (to smell)	58.48	5	CVCCVC	6	P0 P1
Attraper (to catch)	35.32	6	VCCCVCVC	8	P0 P1
Cuisiner (to cook)	11.99	7	CVVCVCVC	8	P0 (P1)
Fumer (to smoke)	35.91	4	CVCVC	5	P0
Marcher (to walk)	85.34	5	CVCCCVC	7	P0 (PL)
Pêcher (to fish)	9.04	4	CVCCVC	6	P0 (P1)
Découper (to cut out)	4.36	6	CVCVVCVC	8	P0 P1

*Note*. FREQUENCY-FILMS: Lexique 3.83, [72]; CV: syllabic structure: Consonant-vowel; P0: avalent verb; P1: monovalent verb; P2: bivalent verb; P3: trivalent verb; PP: verb used with a preposition.

**Table A2:** List of Untrained Verbs.

VERBS	FREQUENCY-FILMS	NUMBERPHONEMES	CV	NUMBERLETTERS	VALENCE
Aider (to help)	362.77	3	VVCVC	5	P0 P1 (PP<with>)
Peigner (to comb)	0.85	4	CVVCCVC	7	P0 P1
Sonner (to ring)	11.77	4	CVCCVC	6	P0 P1
Visser (to screw)	1.45	4	CVCCVC	6	P0 (P1) (PP<on>)
Coudre (to sew)	4.83	4	CVVCCV	6	P0 (P1) (PP<on>)
Clouer (to nail)	1.4	4	CCVVVC	6	P0 P1
Cibler (to target)	5.87	4	CVCVC	5	P0 (PL)
Semer (to sow)	6.09	4	CVCVC	5	P0 P1 (PL)
Jurer (to swear)	7.58	4	CVCVC	5	P0 (P1) (P2)
Coller (to stick)	10.33	4	CVCCVC	6	P0 P1 (PL)
Signer (to sign)	29.25	4	CVCCVC	6	P0 P1
Arroser (to water)	5.53	5	VCCVCVC	7	P0 P1 (P3)
Brancher (to plug in)	4.4	5	CCVCCCVC	8	P0 P1 (PP<on>)
Allumer (to turn on)	11.98	5	VCCVCVC	7	P0 (P1)
Crier (to scream)	31.49	5	CCVVC	5	P0
Écouter (to listen)	73.13	5	VCVVCVC	7	P0 (P1)
Afficher (to put up)	2.06	5	VCCVCCVC	8	P0 P1
Allumer (to switch on)	11.98	5	VCCVCVC	7	P0 (P1)
Plier (to fold)	5.02	5	CCVVC	5	P0 P1
Jongler (to juggle)	0.83	5	CVCCCVC	7	P0 (PP<with>)
Saluer (to greet)	11.85	5	CVCVVC	6	P0 (P1)
Étudier (to study)	26.86	6	VCVCVVC	7	P0 (P1)
Accrocher (to hang)	10.46	6	VCCCVCCVC	9	P0 P1
Mesurer (to measure)	6.63	6	CVCVCVC	7	P0 (P1)
Conduire (to drive)	60.56	6	CVCCVVCV	8	P0 P1 (PL)
Pédaler (to bicycle)	0.37	6	CVCVCVC	7	P0
Chatouiller (to tickle)	0.85	6	CCVCVVVCCVC	11	P0 (P1)
Ratisser (to rake)	1.13	6	CVCVCCVC	8	P0 P1
Modeler (to model)	1.16	6	CVCVCVC	7	P0 P1 (PP<on>)
Dribbler (to dribble)	0.2	6	CCVCCCVC	8	P0 (P1)
Colorier (to color)	0.25	7	CVCVCVVC	8	P0 (P1)
Éternuer (to sneeze)	1.02	7	VCVCCVVC	8	P0
Tricoter (to knit)	1.37	7	CCVCVCVC	8	P0 (P1)
Calculer (to calculate)	3.09	7	CVCCVCVC	8	P0 (P1)
Ausculter (to examine)	0.57	7	VVCCVCCVC	9	P0 P1
Monter (to go up)	85.7	4	CVCCVC	6	P0 (PL)
Grimper (to climb)	7.48	5	CCVCCVC	7	P0 (P2)
Descender (to go down)	65.28	6	CVCCVCCCV	9	P0 (PDL) (PL)
Plonger (to dive)	9.36	5	CCVCCVC	7	P0 P1 PP<into>
Boire (to drink)	142.15	4	CVVCV	5	P0 (P1) (PP<in>)

*Note*. FREQUENCY-FILMS: Lexique 3.83, [72]; CV: syllabic structure: Consonant-vowel; P0: avalent verb; P1: monovalent verb; P2: bivalent verb; P3: trivalent verb; PP: verb used with a preposition.
